# The Effect of Alginate-Based Silver Nanoparticle Films on Young Arugula Plants (*Eruca vesicaria* L. subsp. *sativa*)

**DOI:** 10.3390/molecules31173045

**Published:** 2026-08-30

**Authors:** Miłosz Rutkowski, Gohar Khachatryan, Karen Khachatryan, Lidia Krzemińska-Fiedorowicz, Andrzej Kalisz, Joanna Gil, Adam Florkiewicz, Katarzyna Starzec, Przemysław Petryszak, Paweł Kaszycki, Agnieszka Sękara

**Affiliations:** 1 Centre for Innovation and Research on Prohealthy and Safe Food, University of Agriculture in Krakow, Al. Mickiewicza 21, 31-120 Krakow, Poland; milosz.rutkowski@student.urk.edu.pl; 2 Faculty of Food Technology, University of Agriculture in Krakow, Al. Mickiewicza 21, 31-120 Krakow, Poland; gohar.khachatryan@urk.edu.pl (G.K.); karen.khachatryan@urk.edu.pl (K.K.); lidia.krzeminska@urk.edu.pl (L.K.-F.); adam.florkiewicz@urk.edu.pl (A.F.); 3 Faculty of Biotechnology and Horticulture, University of Agriculture in Krakow, Al. Mickiewicza 21, 31-120 Krakow, Poland; andrzej.kalisz@urk.edu.pl (A.K.); joanna.gil@urk.edu.pl (J.G.); katarzyna.starzec.sd@student.urk.edu.pl (K.S.); przemyslaw.petryszak@urk.edu.pl (P.P.); pawel.kaszycki@urk.edu.pl (P.K.)

**Keywords:** silver nanoparticles, sodium alginate, nanocomposite films, nanoparticle release kinetics, non-target plant responses, rhizosphere microorganisms, silver bioaccumulation, oxidative stress, *Eruca vesicaria*

## Abstract

Biodegradable alginate films containing silver nanoparticles (AgNPs) are being increasingly investigated as active agricultural materials (e.g., antimicrobial mulches) and active food packaging. However, since these materials ultimately degrade in soil after use, assessing their environmental compatibility and potential phytotoxicity upon release is crucial. The aim of this study was to synthesize films containing AgNPs in sodium alginate using xylose as a reducing agent and to determine their effect on culturable rhizosphere microorganisms and selected biochemical parameters in young arugula (*Eruca vesicaria* L. subsp. *sativa*) plants. Alginate films containing three nominal AgNP loadings (50, 100, and 150 mg L^−1^) and a control film without AgNPs were synthesized. The films were cut into square pieces (4 cm^2^) and placed in 0.076 L multipots filled with peat substrate, into which arugula seeds were sown. During the experiment, the abundance of culturable rhizosphere bacteria and fungi was determined, and the young arugula plants were subjected to biochemical analyses. The results showed that the AgNP-containing films did not significantly affect the abundance of bacteria and fungi in the rhizosphere under the conditions tested. The tested films also did not markedly alter the measured parameters in the tissues of young arugula plants, including ascorbic acid, photosynthetic pigments, sugars, dietary protein, and glutathione. However, they reduced phenolic content, altered antioxidant activity, and led to detectable silver accumulation in plant tissues, especially at the highest nominal AgNP loading (150 mg L^−1^). These findings indicate limited but selective biochemical effects during the early growth stage of arugula rather than a complete absence of plant response.

## 1. Introduction

Nanotechnology is a dynamically developing field of science that involves the production, characterization, and application of materials at the nanoscale. Silver nanoparticles (AgNPs) are among the most widely studied metal nanoparticles because of their antimicrobial activity, and they have found applications in medicine, agriculture, and other sectors [1,2,3,4]. Considerable effort is currently being devoted to developing efficient and reproducible methods for synthesizing metal nanoparticles, including physical, biological, and chemical methods. In chemical synthesis, nanoparticles are typically produced by reacting specific metal salts with suitable reducing agents. The choice of a specific reducing agent can strongly influence the nanoparticle shape, size, and physicochemical properties [5,6,7,8,9]. Nanoparticle synthesis commonly involves stabilizing agents, which help limit aggregation and improve dispersion stability. Biopolymers can act as effective and biodegradable stabilizers. Among naturally derived polymers, sodium alginate has been widely studied because of its biodegradability and its structure based on β-D-mannuronic and α-L-guluronic acid residues [10]. However, embedding of AgNPs in sodium alginate-based biopolymer composites requires evaluation of their biological effects, including their potential impact on plants and other components of the environment [11,12]. In the present study, AgNP-loaded alginate films are considered as model active biodegradable materials with potential applications in active packaging and specialised agricultural films, with particular attention to their possible contact with soil during use or after disposal. Recent studies also show that surface capping and functional stabilisation can strongly influence AgNP colloidal stability, optical behaviour and bioactivity [13,14]. In recent years, increasing attention has been paid to the effects of nanomaterials on plants. Leafy vegetables such as arugula (*Eruca vesicaria* L. subsp. *sativa*) are particularly relevant in this context because they may exhibit measurable, multi-level physiological and biochemical responses to nanoparticle exposure. Sayed et al. [15] used arugula as a model plant in agrochemical studies and demonstrated that AgNP treatment increased glucosinolate content due to increased expression of genes involved in their biosynthetic pathways. Vannini et al. [16] reported that exposure of E. sativa seedlings to PVP-coated AgNPs or AgNO_3_ at an equivalent silver concentration of 10 mg Ag L^−1^ resulted in a similar stimulation of root elongation. Although cysteine complexation indicated that this morphological response was largely mediated by released Ag^+^, the proteomic profiles differed substantially between the treatments: only four differentially abundant proteins were common to AgNP- and AgNO_3_-exposed roots, whereas AgNP exposure specifically affected proteins associated with the endoplasmic reticulum and vacuole. These findings suggest that plant responses to AgNPs include both effects related to released silver ions and particle-specific cellular responses. Işıtan et al. [17] used arugula-derived nanosilver in a biodegradable chitosan coating and reported improved antibacterial and mechanical properties, demonstrating a potential materials application of arugula-mediated AgNP synthesis. The increasing use of AgNPs warrants careful evaluation of their fate and biological effects in the environment, including soil and edible plant tissues.

These observations make arugula a suitable model for investigating how AgNPs behave when delivered in a biodegradable alginate matrix rather than as freely dispersed nanoparticles. Although direct application of AgNPs by spraying, seed priming, or foliar treatment may be simpler, embedding nanoparticles in a biodegradable polymer matrix represents a distinct and relevant carrier-based strategy. Alginate is a film-forming, biocompatible, and biodegradable polysaccharide that can stabilize dispersed particles and act as a carrier system. In plant–substrate systems, matrix-based delivery may enable more localized exposure, modulate release dynamics, and reduce sudden exposure peaks compared with direct aqueous nanoparticle application. These differences are important because nanoparticle effects depend strongly on exposure route, bioavailability, and environmental context [18,19]. Therefore, alginate films should be regarded not as a universally superior alternative to direct AgNP application, but as a complementary platform for studying nanoparticle-based materials in biodegradable form. Recent developments in alginate-based environmental technologies have moved beyond conventional beads used for passive immobilization toward engineered multifunctional systems. In whole-cell remediation, such matrices can support microorganisms functioning as active “living catalysts” rather than merely entrapped biomass [20]. The AgNP-loaded films investigated here represent a non-living material system but similarly illustrate the broader transition toward functionally engineered alginate platforms.

Potential applications of AgNP-loaded alginate films include active packaging and specialised biodegradable agricultural materials. Their potential antimicrobial function during use should be considered together with their possible effects after disposal. Antimicrobial activity has previously been reported for related biodegradable AgNP-containing films [5]. In the present study, arugula and culturable rhizosphere bacteria and fungi were used as non-target models to assess plant and microbial responses to the material in a peat-substrate system.

The aim of the study was to prepare sodium alginate-based films containing three nominal loadings of silver nanoparticles (AgNPs) synthesized using xylose as a reducing agent, and to evaluate their early effects on five-week-old arugula plants and the peat substrate rhizosphere. Specifically, the study assessed (I) the successful preparation and basic physicochemical characterization of Ag-containing films, (II) the abundance of culturable bacteria and fungi, and (III) the selected biochemical parameters of five-week-old arugula plants, including silver accumulation and stress-related traits.

## 2. Results

### 2.1. FTIR Spectral Analysis

**Figure 1:**
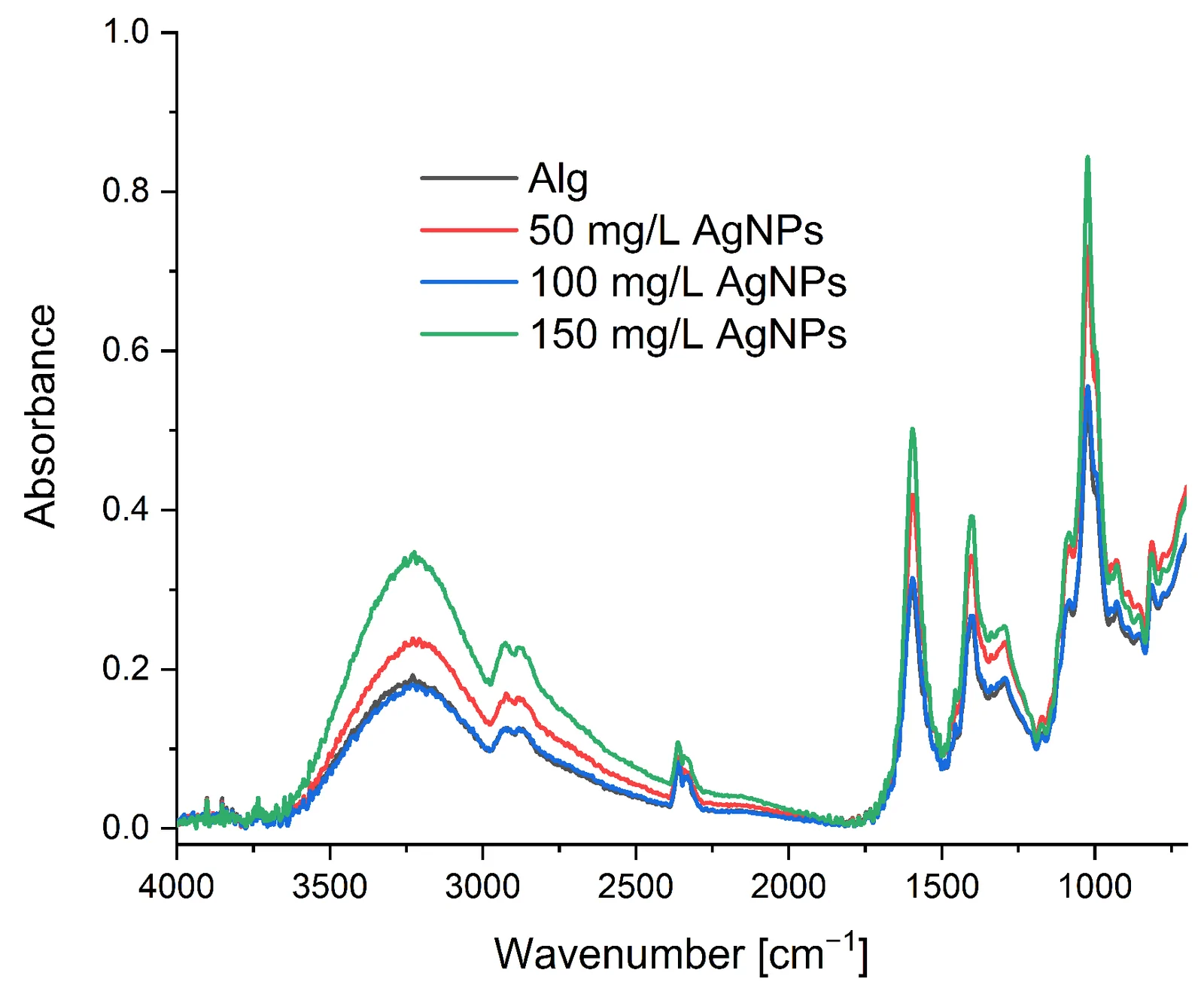
FTIR spectra of control alginate and AgNP-loaded alginate films.

The ATR–FTIR spectra (Figure 1) of the control alginate film (Alg) and the AgNP-loaded films showed the characteristic bands of the alginate matrix. All samples exhibited a broad band in the range 3000–3500 cm^−1^, assigned to the stretching vibrations of hydroxyl (–OH) groups and hydrogen bonding within the hydrophilic polymer network. Bands observed at 2850–2950 cm^−1^ were attributed to C–H stretching vibrations of the polysaccharide backbone. The characteristic alginate carboxylate bands were detected at approximately 1600 cm^−1^ and 1415 cm^−1^, corresponding to the asymmetric and symmetric stretching vibrations of COO^−^ groups, respectively. In addition, intense signals in the 950–1150 cm^−1^ region were associated with C–O and C–O–C stretching vibrations, which are typical of glycosidic bonds and polysaccharide ring structures.

A comparison of the control film with the AgNP-containing films (50, 100 and 150 mg L^−1^) did not reveal any new absorption bands or pronounced shifts in the maxima of the main signals. The differences between the spectra were limited mainly to changes in the intensity of selected bands, most notably within the 3000–3500 cm^−1^ region. These results indicate that the incorporation of AgNPs did not substantially alter the chemical structure of the alginate matrix.

The absence of new bands or major spectral shifts did not provide evidence for the formation of new covalent bonds between the alginate matrix and the nanoparticles. This observation is consistent with predominantly physical incorporation of the nanoparticles into the polymer matrix. Thus, the alginate matrix most likely acted as a carrier for the nanoparticles.

### 2.2. Macroscopic Appearance, UV–Vis Spectroscopy and SEM-Based Particle-Size Analysis of AgNP-Loaded Films

**Figure 2:**
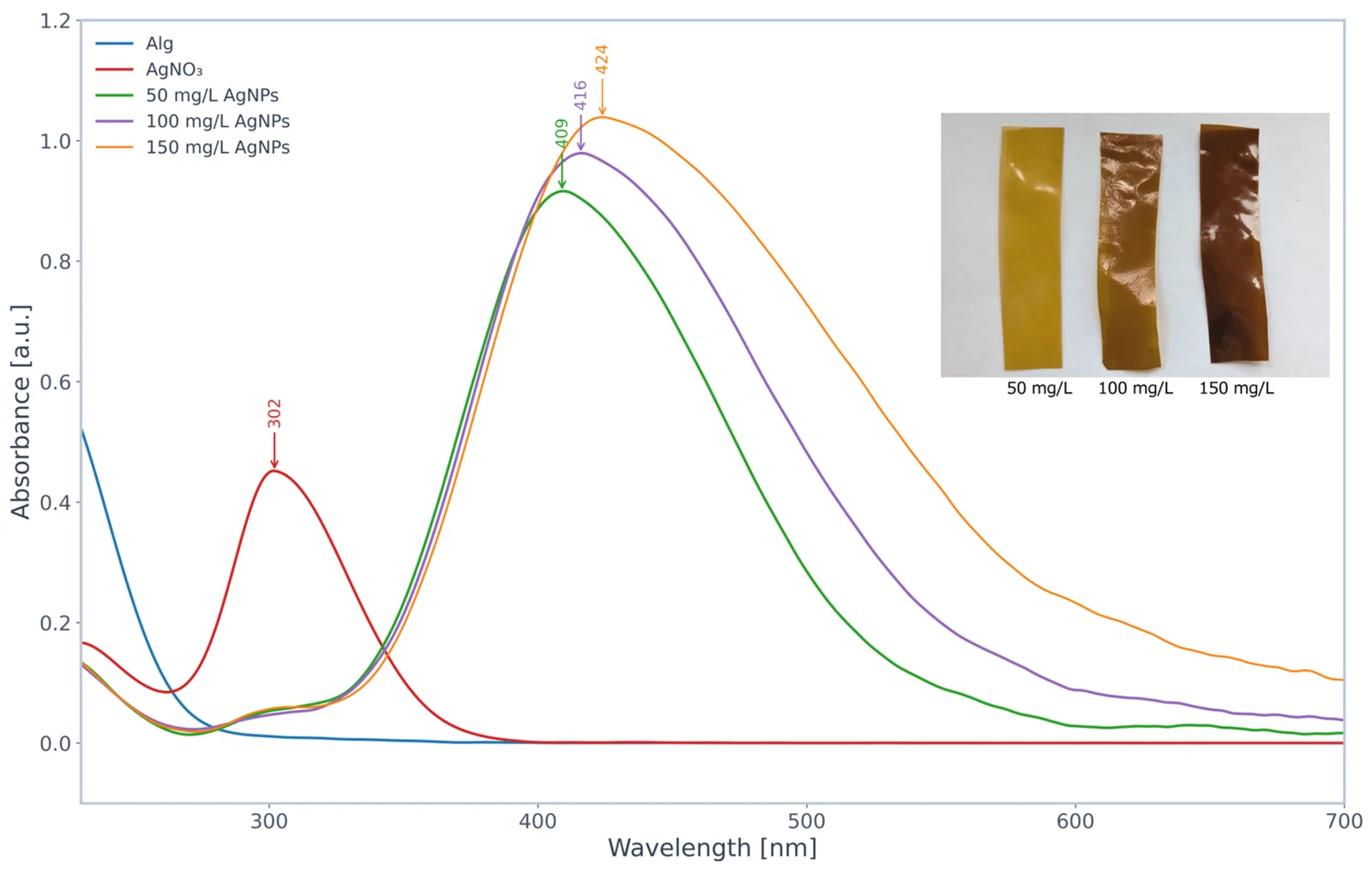
UV–Vis spectra of the control alginate formulation, the silver nitrate precursor, and the AgNP-containing formulations. The inset shows the dried films containing nominal AgNP loadings of 50, 100, and 150 mg L^−1^ (from left to right).

The alginate films containing AgNPs showed visible differences in macroscopic appearance compared with the control film. The control alginate film was transparent and colourless, whereas the AgNP-containing films exhibited progressive yellow-to-brown darkening with increasing nominal AgNP loading. In addition, the films with higher AgNP content showed a more uneven and wrinkled surface (Figure 2).

UV–Vis analysis of diluted alginate gels provided evidence for the formation of silver nanoparticles in the AgNP-containing formulations. In all AgNP-containing samples, a broad absorption band characteristic of silver nanoparticle surface plasmon resonance was observed in the 350–600 nm range, whereas this feature was absent in the control alginate sample. Under the applied measurement conditions, no absorption band attributable to the silver nitrate precursor was observed in the AgNP-containing formulations.

SEM observations revealed clearly distinguishable particle-like objects within the alginate matrix (Table 1). Image analysis of one selected micrograph per formulation identified 36, 60 and 108 measurable objects for films containing nominal AgNP loadings of 50, 100 and 150 mg L^−1^, respectively. Their mean equivalent diameters were 7.42 ± 1.40, 7.93 ± 1.59 and 8.57 ± 1.79 nm, respectively.

**Figure 3:**
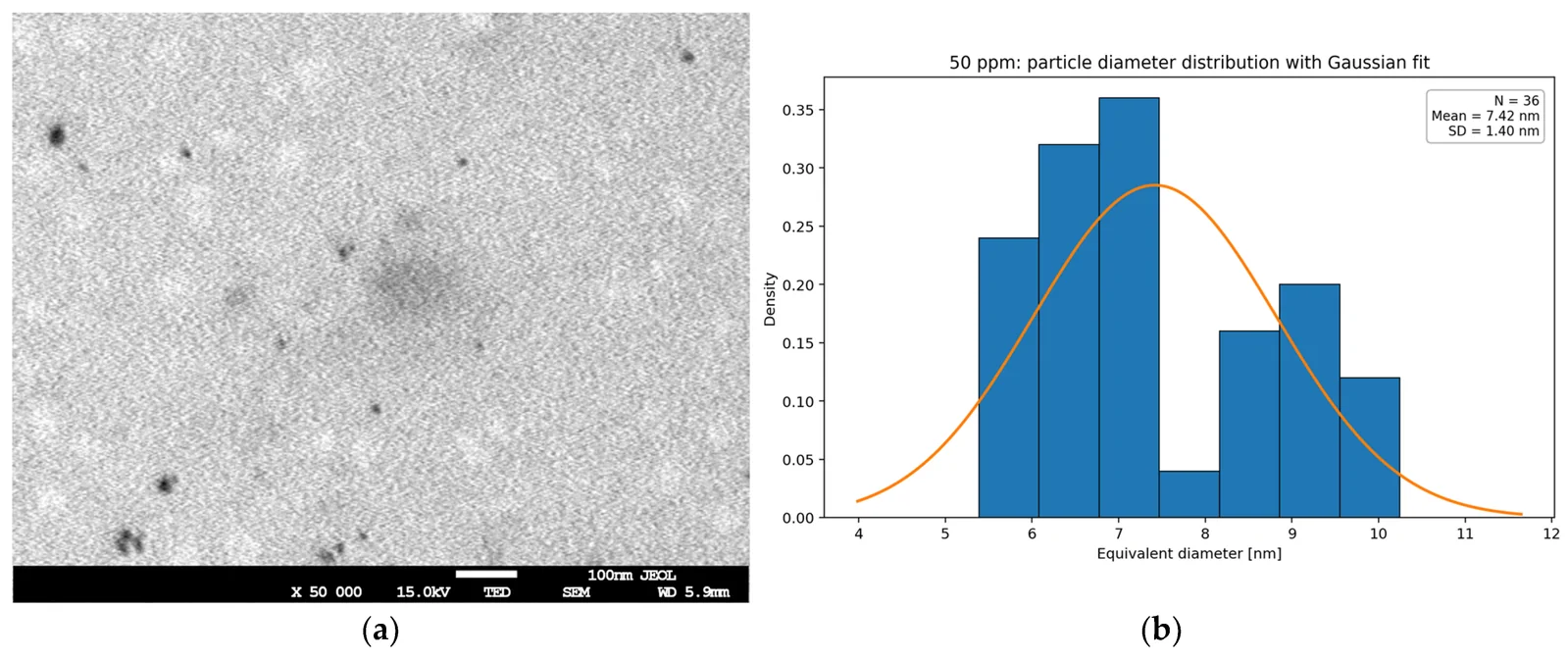

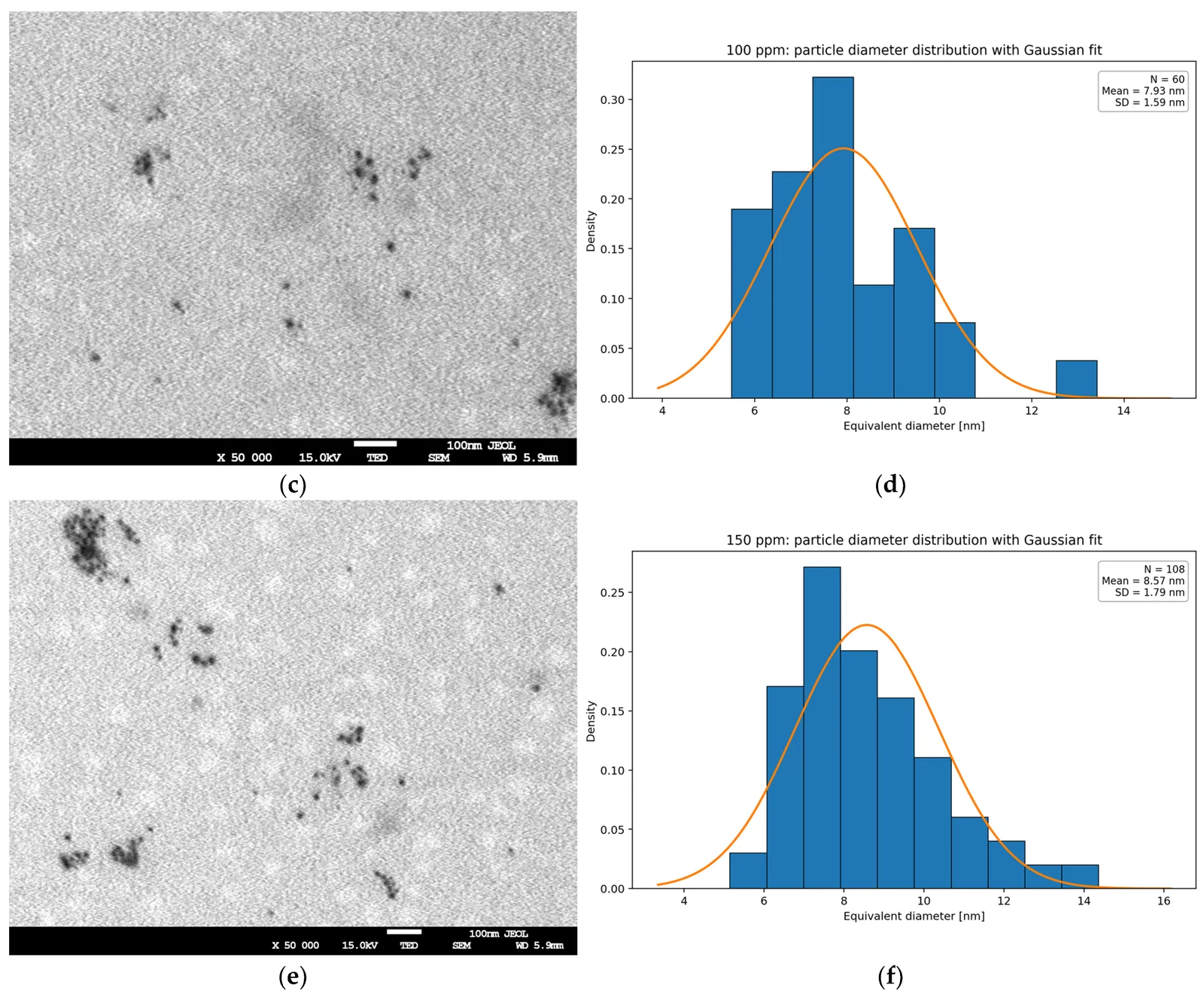
Selected SEM micrographs and corresponding descriptive particle-size histograms for films containing nominal AgNP loadings of 50 mg L^−1^ (**a**,**d**), 100 mg L^−1^ (**b**,**e**), and 150 mg L^−1^ (**c**,**f**). The histograms were based on objects measured in one selected micrograph per formulation. Gaussian curves are shown as visual guides only.

The image-based dispersion index, calculated as (SD/mean)^2^, ranged from 0.036 to 0.044. Mean circularity values ranged from 0.819 to 0.854, mean aspect ratios from 1.94 to 2.06 and maximum Feret diameters from 23.80 to 33.17 nm. These values indicate predominantly compact, but not perfectly spherical, nanoscale objects in the analysed fields of view, with only a slight increase in mean equivalent diameter at higher nominal loading. Because one selected micrograph was analysed for each formulation, the results should be interpreted as field-specific descriptive estimates rather than statistically representative size distributions for the entire films. Together with the UV–Vis results, the SEM observations provide complementary evidence for nanoscale particle-like objects in the AgNP-containing alginate films (Figure 3).

### 2.3. Release Profile and Kinetic Modelling of the Plasmonically Active AgNP-Equivalent Fraction

To better characterise potential exposure, a simplified release experiment was performed for the AgNP-loaded alginate film. For each formulation (50, 100, and 150 mg L^−1^), a 0.1 g piece of dried AgNP-loaded alginate film was immersed in 50 mL of distilled water, and the UV-Vis signal was monitored at the surface plasmon resonance (SPR) band of AgNPs centred at approximately 416 nm. The calibration curve was prepared using a dilution series of freshly synthesised xylose-reduced AgNP gel. The relationship between absorbance and concentration was linear and is described by the equation A_416_ = 0.115621c + 0.001429, with R^2^ = 0.99993, where c is the AgNP-equivalent concentration expressed in ppm. Release was normalised to the formulation-specific theoretical maximum concentration; for the 100 mg L^−1^ formulation, this value was 7.114 ppm.

**Figure 4:**
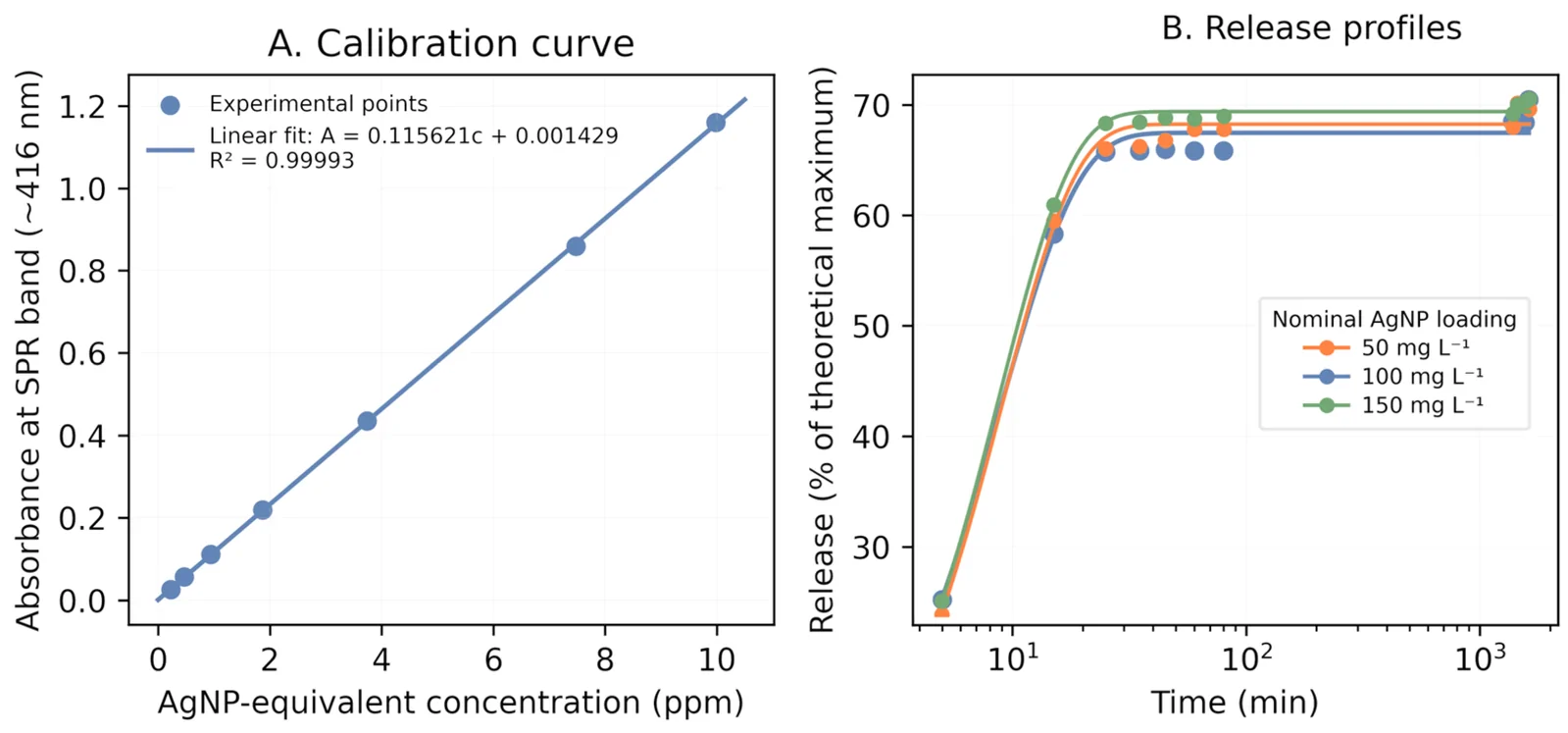
UV–Vis-based characterisation of AgNP release from alginate films. (**A**) Calibration curve prepared from freshly synthesised xylose-reduced AgNP gel using the AgNP SPR band centred at approximately 416 nm. (**B**) Comparative release profiles of the plasmonically active AgNP-equivalent fraction from the 50, 100, and 150 mg L^−1^ formulations, with the corresponding Weibull fits.

The release profile showed a rapid initial phase followed by a slower approach to a quasi-plateau (Figure 4). At 5 min, the released fractions were 23.93%, 25.24%, and 25.12% for the 50, 100, and 150 mg L^−1^ formulations, respectively; after 15 min, the corresponding values were 59.50%, 58.30%, and 60.95%. All three profiles then approached a quasi-plateau. After 1620 min, the released fractions were 69.65%, 70.46%, and 70.57%, corresponding to 4.36, 5.01, and 5.46 ppm AgNP-equivalent for the 50, 100, and 150 mg L^−1^ formulations, respectively.

The release data were fitted using the Weibull model:

$$F\left(t\right)=F_{\infty }{\left[1\;-\;exp(-(t/\tau \right)}^{\beta })]$$

where *F*(*t*) is the released fraction at time *t*, *F_∞_* is the asymptotic released fraction, *τ* is the time-scale parameter, and *β* is the shape parameter. The fitted parameters were as follows: for 50 mg L^−1^, *F_∞_* = 68.27%, *τ* = 9.11 min, *β* = 1.385, R^2^ = 0.9908, and RMSE = 1.23%; for 100 mg L^−1^, *F_∞_* = 67.46%, *τ* = 8.92 min, *β* = 1.30, R^2^ = 0.9865, and RMSE = 1.42%; and for 150 mg L^−1^, *F_∞_* = 69.41%, *τ* = 8.85 min, *β* = 1.400, R^2^ = 0.9975, and RMSE = 0.63%. The close *F_∞_*, *τ*, and *β* values indicate comparable fractional release profiles across the three formulations, characterised by a rapid initial release followed by a slower approach to a quasi-plateau (Table 2).

### 2.4. Abundance of Soil Microorganisms

**Figure 5:**
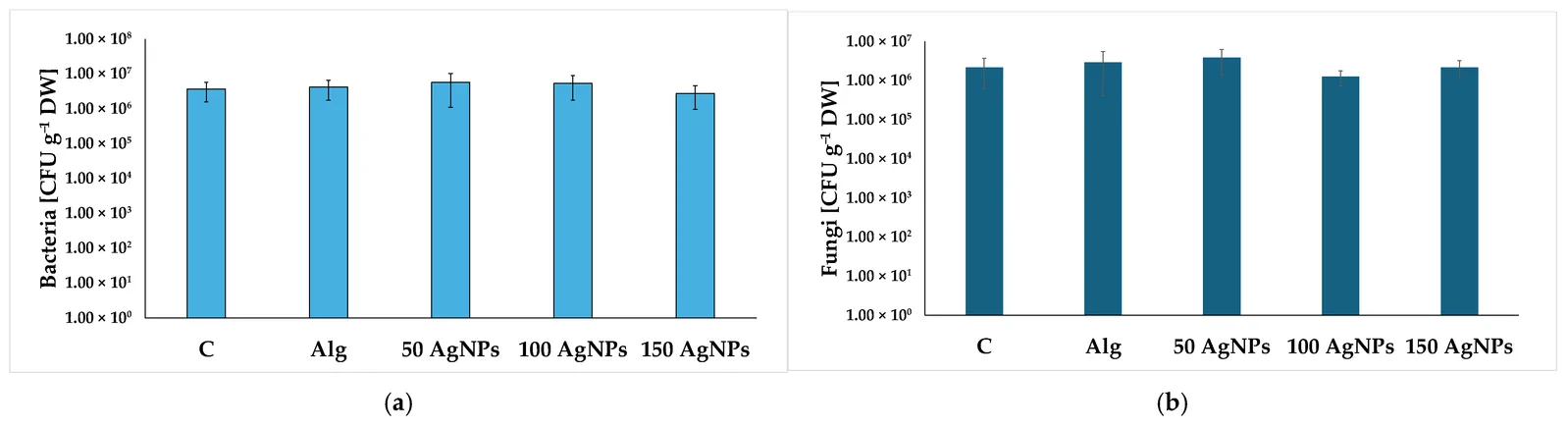
Culturable bacterial (**a**) and fungal (**b**) populations in the peat substrate following treatment with alginate films containing different nominal AgNP loadings. Experimental treatments: C—no film added to the substrate; Alg—alginate film without AgNPs; 50 AgNPs, 100 AgNPs and 150 AgNPs—alginate films with nominal loadings of 50, 100, and 150 mg L^−1^, respectively. Data are presented as means ± standard error (SE), *n* = 5. No letters in the figure indicate no statistically significant differences between treatments.

No significant changes were found in the abundance of culturable rhizosphere bacteria [Figure 5a] or fungi [Figure 5b] after the application of the tested films.

### 2.5. Silver Content and Stress Biomarkers in Plant Tissues

**Figure 6:**
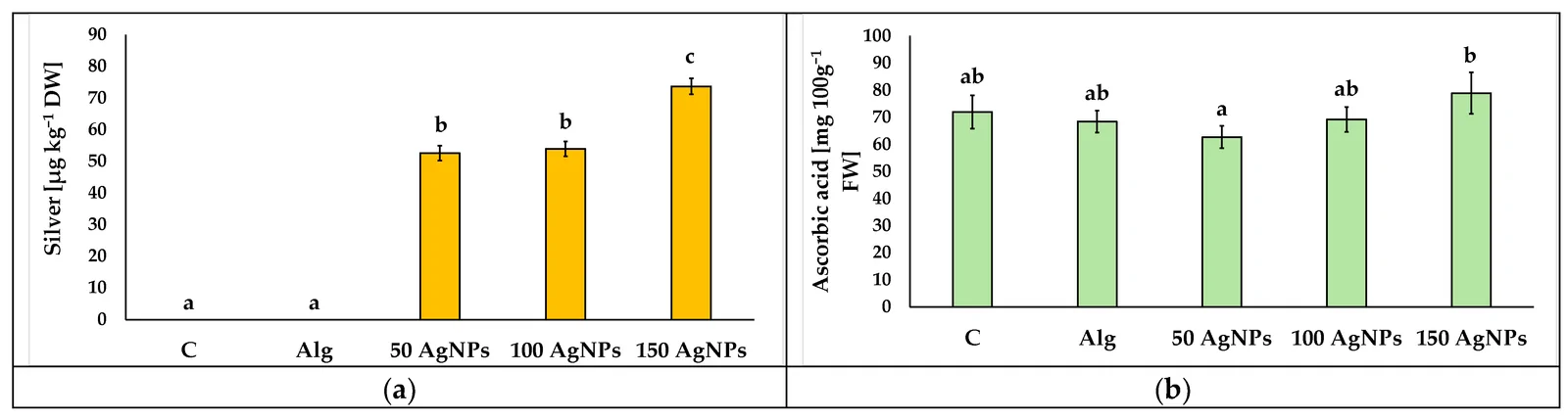

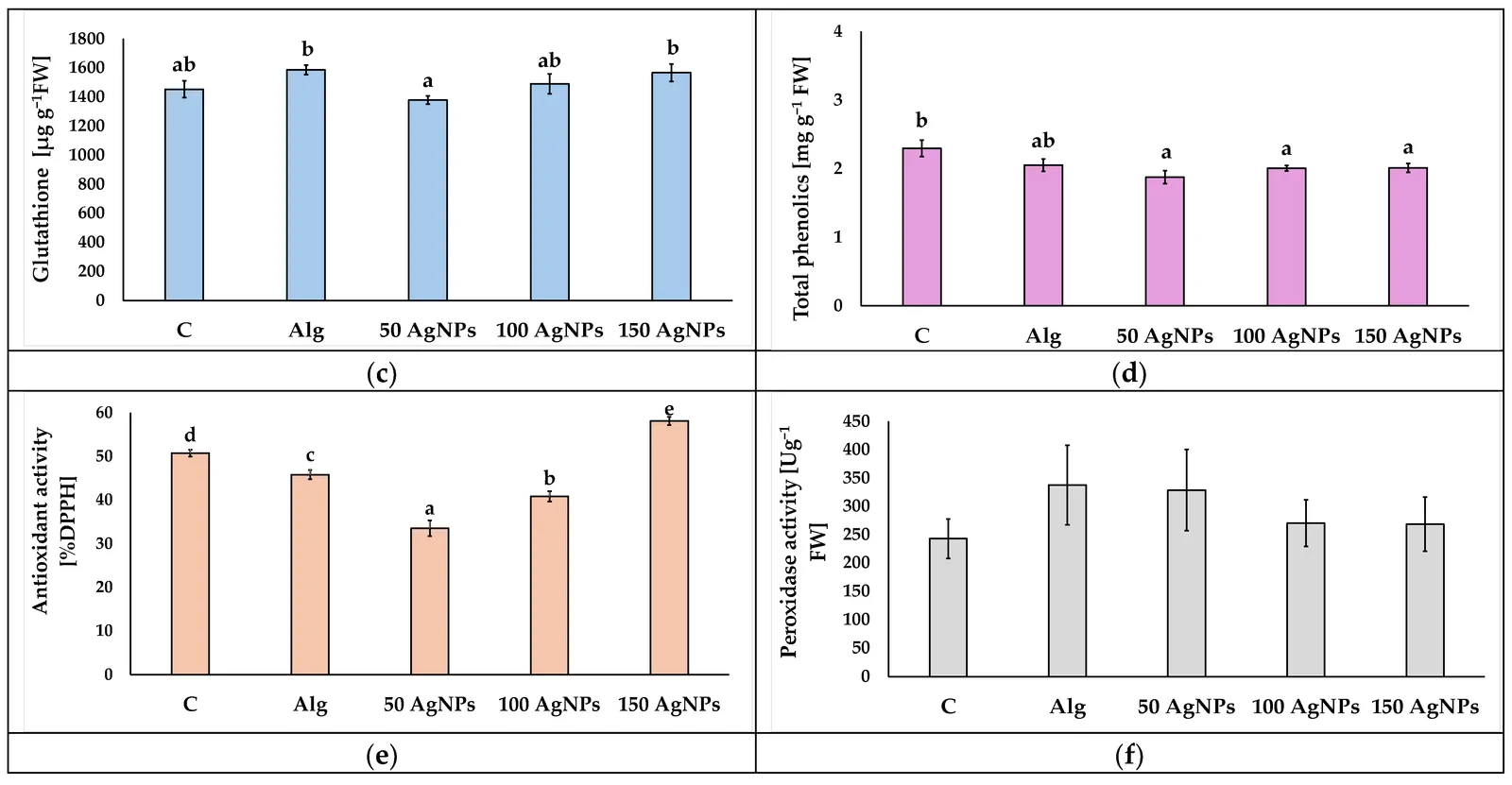
Silver content and stress biomarkers in plant tissues. Analyzed parameters: (**a**)—silver, (**b**)—ascorbic acid, (**c**)—glutathione, (**d**)—polyphenolic compounds, (**e**)—antioxidant activity, (**f**)—peroxidase activity. Experimental treatments: C—no added film to the substrate; Alg—alginate film without AgNPs; 50 AgNPs, 100 AgNPs and 150 AgNPs—alginate films with nominal AgNP loadings of 50, 100, and 150 mg L^−1^, respectively. Statistically, differences marked with letters (a, b, c, d, e) differ significantly at *p* ≤ 0.05 according to Fisher’s LSD test. Data are presented as means ± standard error (SE), *n* = 5. No letters in the figure indicate no statistically significant differences between treatments.

The silver content in plant tissues treated with films containing nominal AgNP loadings of 50 and 100 mg L^−1^ did not differ significantly [Figure 6a]. Only plants treated with the highest nominal AgNP loading (150 mg L^−1^) showed a significantly higher silver content than the control treatments (C, Alg) and the other AgNP treatments. No silver was detected in the tissues of arugula in the control treatments [Figure 6a]. No significant differences in ascorbic acid content were observed between any AgNP treatment and the control treatments. However, plants exposed to 150 mg L^−1^ AgNPs contained significantly more ascorbic acid than those treated with 50 mg L^−1^ AgNPs [Figure 6b]. Glutathione concentrations did not differ significantly between the AgNP treatments and the controls. Nevertheless, plants exposed to 50 mg L^−1^ AgNPs showed significantly lower glutathione levels than those in the Alg control and the 150 mg L^−1^ treatment [Figure 6c]. The content of polyphenolic compounds was significantly lower in plants grown in the presence of all three tested AgNP loadings compared with plants grown under control conditions (C) [Figure 6d]. Arugula exposed to the highest nominal AgNP loading (150 mg L^−1^) showed significantly higher antioxidant activity than all other treatments, whereas plants treated with 50 and 100 mg L^−1^ AgNPs had lower antioxidant activity than the control plants (C, Alg) [Figure 6e]. No significant differences in peroxidase activity were found among treatments [Figure 6f].

### 2.6. Photosynthetic Pigments Content in Plant Tissues

**Figure 7:**
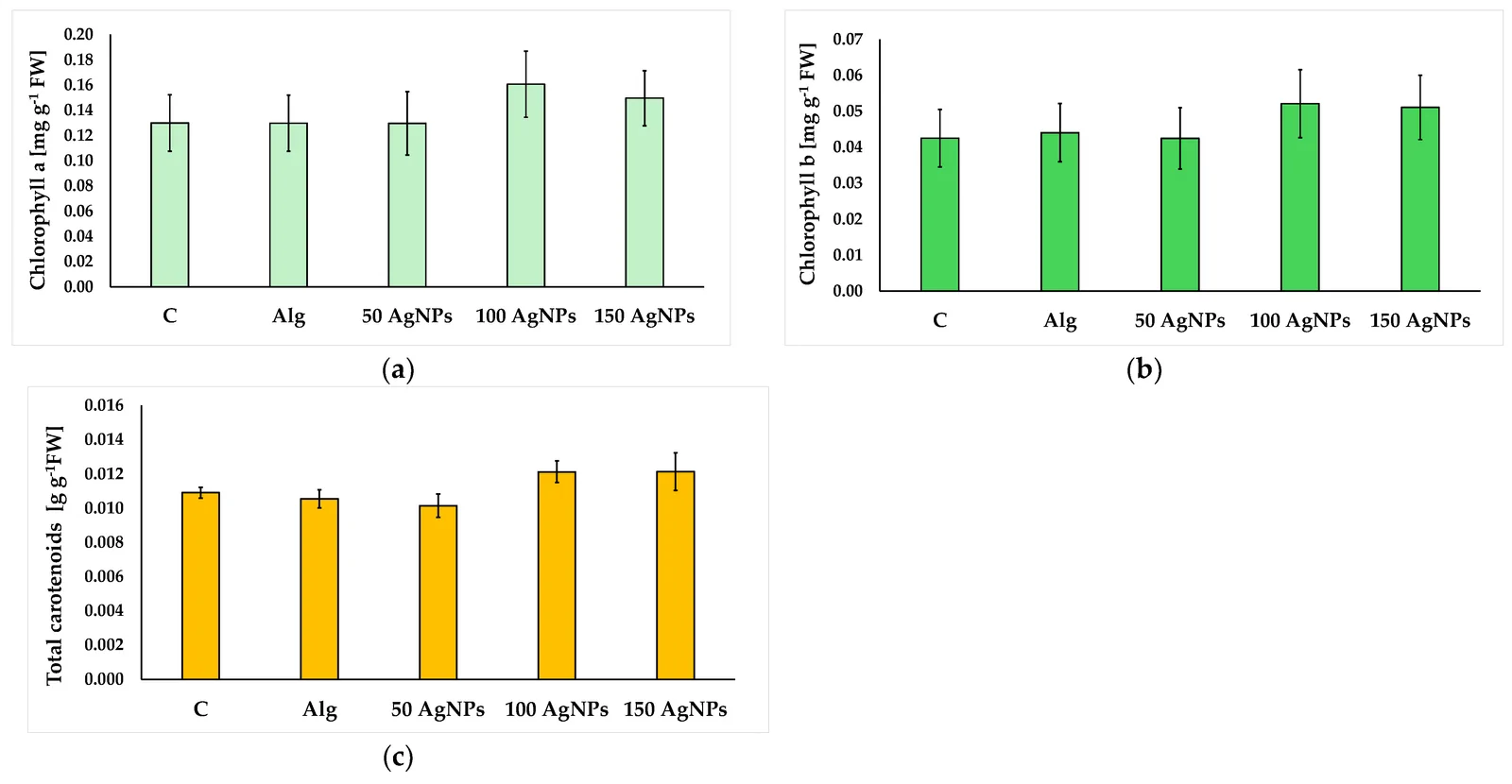
Photosynthetic pigment contents in plant tissues. Analyzed parameters: (**a**)—chlorophyll *a*, (**b**)—chlorophyll *b*, (**c**)—total carotenoids. Experimental treatments: C—no film added to the substrate; Alg—alginate film without AgNPs; 50 AgNPs, 100 AgNPs, and 150 AgNPs—alginate films with nominal AgNP loadings of 50, 100, and 150 mg L^−1^, respectively. Data are presented as means ± standard error (SE), *n* = 5. No letters in the figure indicate no statistically significant differences between treatments.

No statistical differences were observed in the content of chlorophyll *a* [Figure 7a], chlorophyll *b* [Figure 7b], and carotenoids [Figure 7c] among treatments.

### 2.7. Protein, Dietary Fiber and Sugar Content in Plant Tissues

**Figure 8:**
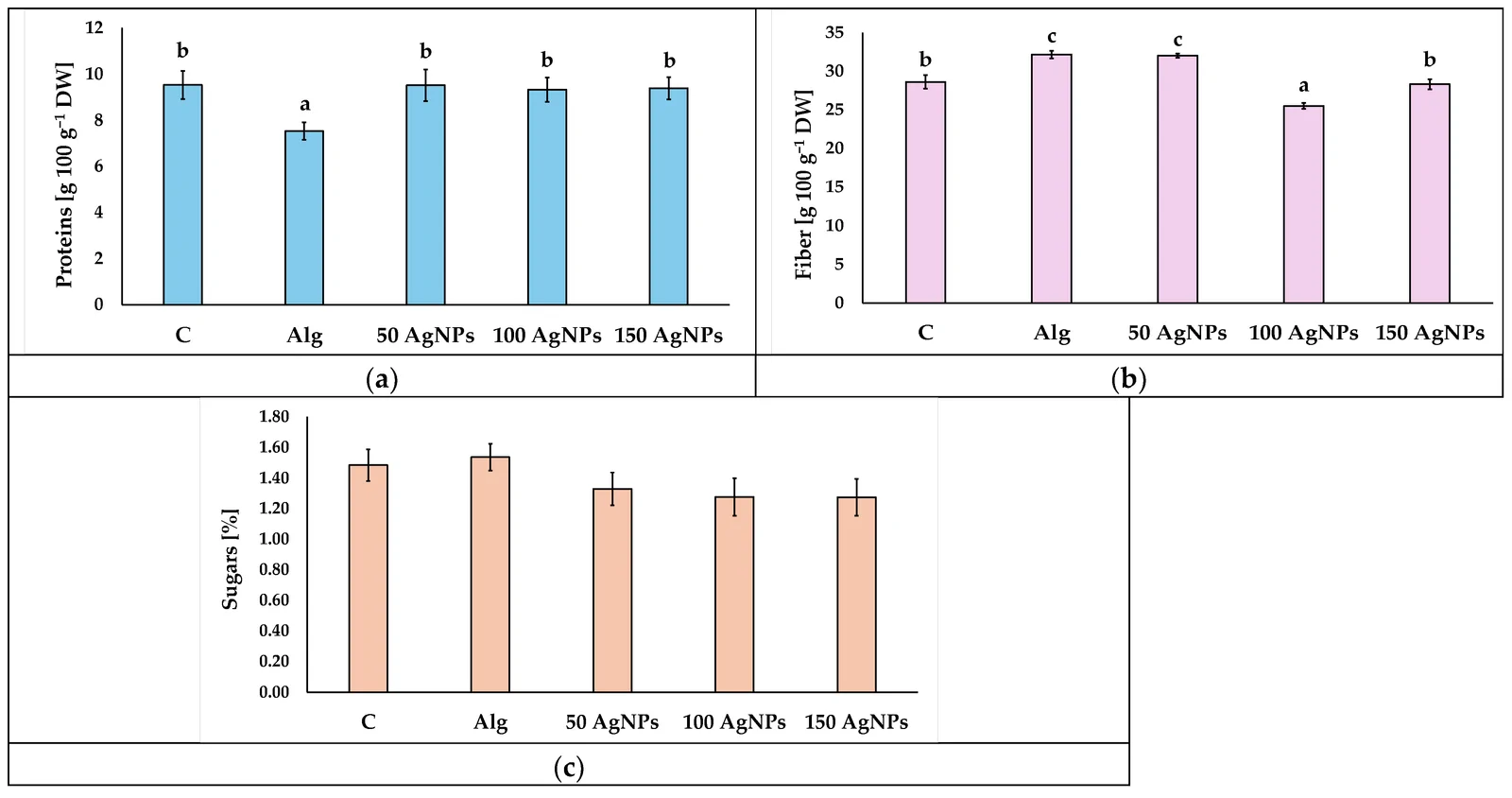
Protein, dietary fiber and sugar content in plant tissues. Analyzed parameters: (**a**)—protein, (**b**)—dietary fiber, (**c**)—total sugar. Experimental treatments: C—no film added to the substrate; Alg—alginate film without AgNPs; 50 AgNPs, 100 AgNPs, and 150 AgNPs—alginate films with nominal AgNP loadings of 50, 100, and 150 mg L^−1^, respectively. Statistically, differences marked with letters (a, b, c) differ significantly at *p* ≤ 0.05 according to Fisher’s LSD test. Data are presented as means ± standard error (SE), *n* = 5. No letters in the figure indicate no statistically significant differences between treatments.

The presence of a sodium alginate film without AgNPs (Alg) caused a significant reduction in dietary protein content compared with the other treatments [Figure 8a]. The presence of the 50 mg L^−1^ AgNP film and the sodium alginate film without AgNPs (Alg) led to a significant increase in dietary fiber content in the plant material compared with the control treatment (C) as well as with the other tested AgNP treatments. Only the 100 mg L^−1^ AgNP treatment significantly reduced dietary fiber content compared with all other treatments [Figure 8b]. The total sugar content did not change significantly among treatments [Figure 8c].

## 3. Discussion

The use of eco-friendly reducing agents and biocompatible carriers is consistent with current efforts to develop more sustainable AgNP-based materials, in accordance with the principles of green chemistry. Variability in nanoparticle size, shape, and biological activity may depend on the reducing system used during synthesis [5]. In our study, the synthesis of silver nanoparticles in sodium alginate using xylose as a non-toxic reducing agent was consistent with previously published studies, owing to the similar reaction conditions, the biological nature of the polymer matrix and the strong reducing properties of the sugar used. This interpretation is supported by the physicochemical characterisation performed in the present study. FTIR analysis indicated that the chemical structure of the alginate matrix remained largely unchanged after nanoparticle incorporation, which suggests that AgNPs were embedded mainly by physical entrapment rather than covalent bonding. The lack of significant spectral shifts or new absorption bands provides evidence regarding the structural nature of the AgNP-loaded films and supports the view that sodium alginate acted as a carrier and temporary reservoir for the nanoparticles. Under moist soil conditions, matrix swelling and gradual degradation could facilitate the release of silver species from the films. This interpretation is consistent with the silver accumulation detected in arugula tissues, particularly at the highest nominal AgNP loading (150 mg L^−1^), and indicates that at least part of the incorporated silver became bioavailable during the 5-week experiment. In addition, UV–Vis spectra confirmed nanoparticle formation and did not show the characteristic signal of the silver nitrate precursor in the freshly prepared AgNP-containing formulations under the applied measurement conditions, suggesting that residual free precursor-derived ionic silver was not detectable in the initial films. However, this does not exclude the possible generation of dissolved silver species during soil exposure as a result of partial oxidative dissolution of AgNPs. Therefore, the present study demonstrates the effects of AgNP-loaded alginate films under nominal loading conditions, but it does not distinguish unequivocally between the contributions of nanoparticulate and dissolved silver during plant exposure. Accordingly, the observed biological responses should be attributed to exposure to the AgNP-loaded alginate material and the silver species released from it collectively, rather than to nanoparticulate silver alone. Soil microorganisms play an important role in plant functioning, and nanoparticle exposure may affect rhizosphere communities. The composition and abundance of rhizosphere microorganisms depend on the plant root system and substances released by plants into the rhizosphere [20,21]. AgNPs may enter soil as a result of agricultural use [21]. In our study, no significant changes were observed in the abundance of culturable rhizosphere microorganisms following exposure to alginate films containing AgNPs. This suggests that, under the tested conditions, the alginate films containing AgNPs did not exert a strong inhibitory effect on these microbial groups. One possible explanation is limited silver bioavailability or gradual release from the film matrix; however, this was not directly measured. Because only culture-dependent abundance was assessed, potential changes in microbial activity or community structure cannot be excluded.

The SEM image analysis yielded mean equivalent diameters of 7.42–8.57 nm across the three formulations. Similar image-based dispersion indices and circularity/aspect-ratio values suggest that increasing the nominal AgNP loading did not produce a pronounced change in the size heterogeneity or overall shape of the objects observed in the selected fields of view. The slightly greater mean equivalent diameter and maximum Feret diameter recorded for the 150 mg L^−1^ formulation may reflect a greater contribution from locally associated or elongated objects at the highest nominal loading. However, because only one selected micrograph per formulation was analysed, these observations are descriptive and do not demonstrate a statistically confirmed loading-dependent change in particle morphology.

The limited effects observed in the present study should not be interpreted as a general absence of biological activity of AgNP-containing materials. Antimicrobial activity has been reported previously for related biodegradable AgNP-containing films [5]. The present work addressed a different question: the response of non-target organisms under plant–substrate exposure conditions. No marked inhibition of culturable bacterial or fungal abundance was detected, whereas plant responses were selective and depended on the measured endpoint and nominal loading. These findings support further assessment of the environmental compatibility of the material but do not establish comprehensive environmental safety.

The aqueous release experiment characterises the initial release behaviour of the representative 100 mg L^−1^ formulation under fully aqueous conditions and does not establish long-term stabilisation or sustained release in peat. The release kinetics of nanosilver from an alginate matrix is determined by multiple factors, including the physicochemical properties of the matrix, its hydration and dispersion behaviour, the colloidal stability of AgNPs, and environmental conditions. In soil-based systems, interpretation is particularly complex because silver release may be accompanied by aggregation, adsorption onto substrate components, complexation with organic matter, and partial oxidative dissolution of AgNPs to Ag^+^ [21].

In the present work, the aqueous release test was used as a simplified model. Its purpose was not to provide complete silver speciation in the substrate, but to describe the release profile of the AgNP fraction retaining plasmonic activity. The rapid initial release phase can be attributed mainly to hydration, swelling and partial dispersion of the alginate film, whereas the subsequent quasi-plateau suggests that part of the silver fraction remained retained within the polymer matrix or became less readily available for optical detection.

The simplified aqueous test was not intended to reproduce the complete five-week exposure in peat. It showed the rapid appearance of an optically detectable, plasmonically active AgNP-equivalent fraction during film hydration. The quasi-plateau reached within approximately 27 h describes behaviour under fully aqueous conditions. Release in peat may differ because of lower and fluctuating water availability, adsorption to substrate components, aggregation and silver dissolution. Because release kinetics and silver speciation were not measured in the peat substrate, no conclusion can be drawn regarding sustained nanoparticle release throughout the full plant-growth period. If prolonged antimicrobial exposure in the substrate is required, the rapid initial aqueous release observed here indicates that further formulation optimisation would be necessary. The Weibull equation was used as an empirical function to describe the release profile [22,23,24]. In the present study, the model provided a good fit to the three experimental profiles and allowed the release process to be described using interpretable parameters: F∞ as the asymptotic plasmonically active AgNP-equivalent fraction, τ as the characteristic time scale, and β as the curve-shape parameter. The Weibull function was retained as an a priori empirical descriptor that provides a common parameterisation of the rapid-rise-to-plateau profiles across the three formulations; it was not selected through a comparative model-ranking procedure.

However, UV-Vis monitoring at the SPR band around 416 nm provides information on the plasmonically active AgNP-equivalent fraction rather than total silver release. This method cannot distinguish between intact AgNPs, aggregated AgNPs, Ag^+^ ions, or matrix- and synthesis-derived components absorbing in the UV region. This limitation is particularly relevant because the AgNPs were obtained by xylose-mediated reduction of AgNO_3_, immobilised in an alginate matrix, and dried to form films. During drying, storage, rehydration and substrate exposure, the colloidal state of the nanoparticles may change, and partial aggregation or oxidative dissolution cannot be excluded. Thus, the value of 70.46% after 1620 min should be interpreted as a UV-Vis signal corresponding to the plasmonically active AgNP-equivalent fraction, not as evidence that the same proportion of all nanoparticles remained unchanged. Complete discrimination between nanoparticulate, ionic and matrix-related effects would require additional experiments, including an AgNO_3_ ionic silver control, total silver determination in the eluate by AAS/ICP-OES/ICP-MS, operational separation of dissolved and colloidal fractions, and DLS/zeta-potential characterisation of the released material [25].

AgNP exposure may induce plant physiological responses depending on species, concentration, and exposure route [26]. In our study, determination of silver content confirmed that the application of films with AgNPs resulted in the presence of silver in the tissues of five-week-old arugula plants, whereas no silver was detected in the control plants. The use of a film with the highest nominal AgNP loading (150 mg L^−1^) resulted in a significantly higher silver content compared with the lower nominal loadings. This observation is consistent with the release of silver from the alginate matrix and its subsequent uptake by plants. The accumulation detected in plant tissues indicates that at least part of the incorporated silver became bioavailable under the experimental conditions. However, the present study did not include an ionic silver control and did not quantify dissolved silver during exposure; therefore, it cannot disentangle the relative contributions of particulate and dissolved silver to the observed responses. It is possible that the amount of silver entering the plant tissues from the films containing 50 and 100 mg L^−1^ AgNPs was similar enough to result in comparable statistical values. Jurkow et al. [27] found that foliar application of AgNPs did not affect ascorbic acid or glutathione content in oak-leaf lettuce (*Lactuca sativa* L.), although they observed increased peroxidase activity. In the same study, an increase in polyphenolic compounds and elevated antioxidant activity were observed after foliar application of AgNPs at 40 mg L^−1^. An et al. [28] documented stimulation of ascorbic acid content after coating asparagus (*Asparagus officinalis* L.) spears with AgNPs. Homaee and Ehsanpour [29] did not observe an increase in glutathione content in potato (*Solanum tuberosum* L.) after treatment with AgNPs under in vitro conditions, whereas Najafi et al. [30] reported stimulation of polyphenolic compounds in wheat (*Triticum aestivum* L.) seedlings exposed to AgNPs. In our study, no stimulation of peroxidase activity and no increase in ascorbic acid or glutathione content were observed after the application of tested AgNP loadings. However, we found a significant reduction in polyphenolic compounds after exposure to all tested AgNP concentrations. Antioxidant activity decreased at 50 and 100 mg L^−1^ AgNPs, compared with the control, whereas treatment with 150 mg L^−1^ of AgNP-containing film significantly increased antioxidant activity. AgNP exposure does not necessarily result in a uniform activation of antioxidant defenses in plants. Instead, published studies show that enzymatic and non-enzymatic antioxidant responses may increase, decrease, or remain unchanged depending on plant species, developmental stage, nanoparticle properties, dose, exposure route, and duration of exposure [30,31]. Therefore, the unchanged peroxidase activity observed in the present study should not be considered unusual, but rather interpreted as part of a selective, endpoint-specific response pattern to AgNP exposure. In addition, sodium alginate without nanosilver did not significantly affect the levels of ascorbic acid, glutathione, and peroxidase activity, although it reduced antioxidant activity compared with the control treatments. Thus, our results should be interpreted not as the absence of plant response, but rather as evidence of a selective and dose-dependent biochemical response to AgNP exposure during early growth. At the same time, the lack of significant changes in several other parameters suggests that these responses did not result in broad adverse changes across the measured biochemical parameters.

Photosynthetic pigments are important for the functioning of plants and their content can be affected by nanoparticle treatment. Alhammad et al. [32] demonstrated an increase in chlorophyll *a*, chlorophyll *b*, and carotenoid content in faba bean (*Vicia faba* L.) after seed priming with silver nanoparticles at a concentration of 10 mg L^−1^, whereas treatment with a higher AgNP loading (50 mg L^−1^) resulted in a decrease in the carotenoid content. Mirzajani et al. [33] reported increased carotenoid content in rice (*Oryza sativa* L.) after exposure to nanoparticles at a concentration of 60 mg L^−1^. Hasan [34] found increased chlorophyll *a* and *b* content in hydroponically grown lettuce (*Lactuca sativa* L.) at lower concentrations of AgNPs (25 and 50 mg L^−1^), whereas chlorophyll content decreased at 100 mg L^−1^ of AgNPs. Tomaszewska-Sowa [35] reported a significant decrease in photosynthetic pigments after the application of silver nanoparticles in rapeseed (*Brassica napus* L.), while Aly [36] observed increased pigment accumulation in lettuce (*Lactuca sativa* L.) under the influence of alginate. In the present work, we found no significant effect of AgNPs at any of the three tested loadings on the chlorophyll *a*, chlorophyll *b*, and total carotenoid concentrations. Thus, pigment accumulation appeared relatively stable under the tested treatments at the analysed growth stage.

Proteins participate in the cellular response to abiotic stress factors [37]. Sadak et al. [38] documented an increase in protein content in fenugreek following treatment with AgNPs, and increased protein content was also reported in studies on beans (*Phaseolus vulgaris* L.) and corn (*Zea mays* L.) treated with AgNPs [39]. In our study, the tested AgNP nominal loadings did not significantly affect the protein content in arugula. Only the presence of sodium alginate without AgNPs (Alg) decreased the protein content. This suggests that protein accumulation was less responsive than some other biochemical endpoints under the present conditions.

Dietary fiber is a polysaccharide present in plant-derived foods, including vegetables [40]. Our results show that the presence of 50 mg L^−1^ AgNP film and sodium alginate alone (Alg) increased dietary fiber content in arugula seedlings, whereas 100 mg L^−1^ AgNP treatment reduced the fiber content compared with the control (C). The 150 mg L^−1^ AgNP treatment did not significantly affect the dietary fiber content. This non-monotonic response suggests that the effect was treatment-specific rather than clearly dose-dependent. The increase in dietary fiber observed in the presence of sodium alginate alone may reflect matrix-related effects rather than a direct action of silver. Because no measurements of cell-wall metabolism, carbon allocation, or silver kinetics were performed, the mechanisms responsible for these differences remain unclear. Therefore, these findings should be interpreted as treatment-associated effects rather than evidence of a specific metabolic mechanism.

Carbohydrates play roles in cellular respiration, photosynthesis, and the synthesis of numerous defense compounds. Sugars are also signalling molecules involved in abiotic stress-induced gene expression [41,42]. Stałanowska et al. [43] observed an increase in sugar content in pea (*Pisum sativum* L.) and wheat (*Triticum aestivum* L.) after exposure to ZnO nanoparticles. In our study, treatment with AgNP-containing alginate films did not affect the sugar content in arugula, and sodium alginate without nanosilver also did not increase sugar accumulation in plant tissues. Overall, the results show that alginate-embedded AgNPs induced selective responses in five-week-old arugula plants and did not produce a uniform pattern of adverse changes across the measured endpoints. Related alginate-based and suspension-based AgNP systems have produced strongly context-dependent plant responses. In cucumber seedlings, alginate gels containing 10 mg L^−1^ AgNPs caused limited growth effects, whereas exposure to 20 mg L^−1^ inhibited root and shoot growth and modified stress-related biochemical parameters [10]. In rice, freely dispersed AgNPs stimulated root growth at concentrations up to 30 mg L^−1^ but restricted growth at 60 mg L^−1^ and altered rhizosphere bacterial populations [33]. In comparison, the solid-film exposure used in the present peat-based experiment did not cause broad adverse changes across the measured endpoints or detectable changes in the abundance of culturable bacteria and fungi, although silver accumulation and selective biochemical responses were observed. Direct quantitative comparison among these studies remains limited because nanoparticle properties, exposure matrices, application routes, durations, and dose metrics differed.

The present results show the early biochemical and microbiological response profile of a biodegradable AgNP carrier system in a plant–substrate model. Such information is important for assessing the biological compatibility of carrier-based nanomaterials before considering their potential agricultural use. The practical significance of these findings lies in the preliminary environmental compatibility assessment of the AgNP-loaded carrier rather than in demonstrating a plant-growth-promoting effect. The absence of broad adverse changes in the measured endpoints did not exclude silver uptake or selective biochemical responses in the edible plant. Therefore, the present results do not justify field application of the material; further development would require confirmation of its efficacy against defined target microorganisms, determination of the silver mass balance and speciation, evaluation of long-term release under substrate conditions, and broader assessment of non-target organisms. Further studies should also directly address silver-release kinetics, silver speciation, plant uptake pathways, and rhizosphere community composition in order to explain the mechanisms underlying the observed responses. Several limitations should be considered when interpreting these results. The Alg control was not composition-matched for xylose and the other synthesis components introduced with the silver/ammonia solution, so formulation-dependent effects cannot be attributed solely to AgNPs. Film thickness is reported in Section 4.1, while mechanical properties were not measured; the theoretical dry-solids loading per unit area was calculated from the formulation composition and casting area. Release profiles were determined for all three AgNP-containing formulations under the same simplified aqueous protocol. Conventional whole-plant endpoints such as germination, survival, root and shoot growth and fresh or dry biomass were not quantified; consequently, this study should be regarded as a preliminary non-target plant and rhizosphere response assessment rather than a complete phytotoxicity or environmental-safety evaluation. Total Ag in the dried films was not experimentally determined; therefore, the Ag mass per 4 cm^2^ film piece is reported as a calculated nominal value rather than as an analytical determination.

Future material development may include more actively engineered carrier architectures and controlled porosity to tune matrix hydration, mass transport, and silver release while preserving the biodegradable character of the material. For example, Jin et al. [44] used NaHCO_3_ as a pore-forming agent in a polyvinyl alcohol/sodium alginate carrier, thereby increasing the average pore diameter and improving mass-transfer efficiency. Although that system was designed for the immobilization of aerobic denitrifying bacteria and differs from the AgNP-loaded alginate films investigated here, it demonstrates how porogen-assisted matrix design can alleviate transport limitations associated with dense polymer networks. Similar approaches could be explored in future studies to regulate the hydration and release behaviour of alginate-based nanoparticle carriers.

## 4. Materials and Methods

### 4.1. Synthesis of Silver Nanoparticles in Alginate Films

Chemical reagents used during the chemical synthesis of silver nanoparticles in sodium alginate: silver nitrate (99.99%, Sigma Aldrich, Poland, PubChem CID: 135191), sodium alginate (alginic acid sodium salt, product No. 180947, CAS 9005-38-3, low-viscosity grade, marine-algae origin; Sigma Aldrich, Poznań, Poland), xylose (Sigma Aldrich, Poznań, Poland), glycerol (99.5%, Sigma Aldrich, Poznań, Poland).

According to the manufacturer, this sodium alginate has a viscosity of 15–30 cP for a 1% solution in water and a published molecular-weight range of 12,000–80,000 Da. The manufacturer also reports an approximate monomer composition of 61% mannuronic acid (M) and 39% guluronic acid (G), corresponding to an M/G ratio of approximately 1.56; this composition is an approximate product value rather than a batch-specific specification.

The synthesis reaction of AgNPs in alginate films was performed according to Rutkowski et al. [5,45] with modifications. Alginate gel (1.5%) was obtained by dissolving 12 g of polysaccharide in 788 g of deionised water at 80 °C for 24 h. The process was carried out until a homogeneous suspension was formed. Glycerol was added as a plasticizer in a weight ratio of 1:2 (*w*/*w*) to sodium alginate and further mixed for 30 min. The resulting gel was divided by mass into four equal parts of 200 g each. The silver-containing solution, consisting of 10 mL of 0.1 M AgNO_3_, 70 mL of deionized water, and 20 mL of 20% ammonia solution, was added by mass to the first, second, and third alginate samples. The silver-containing solution was added at 8.44, 16.88, and 25.32 g to the 50, 100, and 150 mg L^−1^ formulations. A 4% xylose solution was added dropwise to the three Ag-containing samples in amounts of 13.67, 27.35 and 41.02 g, respectively. To prepare the control alginate film without AgNPs, deionised water was added instead of the silver-containing and reducing solutions, and stirring was continued at 80 °C. The reaction was continued until a yellow colour, characteristic of AgNPs, was obtained. The gels were then poured onto degreased trays and dried at 40 °C for 72 h. Each 200 g formulation was cast onto a 20 × 30 cm tray (600 cm^2^). Based on the principal non-volatile film-forming components (sodium alginate, glycerol, and xylose), the calculated theoretical dry-solids loadings were 7.50, 8.41, 9.32, and 10.23 mg cm^−2^ for the Alg, 50, 100, and 150 mg L^−1^ formulations, respectively. The mean thicknesses of the dried films were 80.3 ± 0.2 µm for Alg and 83.5 ± 0.3, 84.4 ± 0.2, and 85.7 ± 0.2 µm for the films with nominal AgNP loadings of 50, 100, and 150 mg L^−1^, respectively. As a result, biopolymer films with three loadings of silver nanoparticles were obtained: 50, 100, and 150 mg L^−1^, as well as a control film without AgNPs (Alg) [Table 3].

The morphology and size of particle-like objects in the AgNP-containing films were evaluated by scanning electron microscopy (SEM) using a JEOL JSM-7500F microscope (JEOL, Tokyo, Japan) equipped with a transmission electron detector (TED). Micrographs were acquired at 50,000× magnification and 15.0 kV. One selected micrograph per formulation was analysed using ImageJ software (version 1.54f, National Institutes of Health, Bethesda, MD, USA). Clearly distinguishable objects were included in the analysis, whereas large unresolved agglomerates, overlapping objects and objects intersecting the image borders were excluded. Equivalent diameter, circularity, aspect ratio and maximum Feret diameter were determined. The value *n* denotes the number of objects included in the analysis and does not represent nanoparticle concentration in the film. Particle-size histograms and descriptive Gaussian curves were generated using OriginPro software (Origin 2015 (9.2), OriginLab Corporation, Northampton, MA, USA). Because one field of view was analysed per formulation, the resulting values were treated as field-specific descriptive image-analysis estimates.

Alginate gels were diluted ten-fold with distilled water prior to UV–Vis measurements. UV–Vis absorption spectra were recorded over the range 200–700 nm using a Shimadzu 2101 scanning spectrophotometer (Shimadzu, Kyoto, Japan) in a quartz cuvette, with distilled water as the reference.

For attenuated total reflectance Fourier-transform infrared (ATR–FTIR) analysis, spectra of the control sample and the films containing silver nanoparticles were recorded over the range 4000–700 cm^−1^ at a resolution of 4 cm^−1^. ATR–FTIR measurements were carried out using a MATTSON 3000 FT-IR spectrophotometer (Madison, WI, USA) equipped with a 30SPEC 30° reflective accessory and a MIRacle ATR accessory (PIKE Technologies Inc., Madison, WI, USA).

In the present study, the values of 50, 100 and 150 mg L^−1^ refer to the nominal AgNP loadings used during film preparation. These values should therefore be interpreted as formulation levels in the alginate films rather than as directly measured silver concentrations in the peat substrate. The macroscopic appearance of the dried films was documented photographically to compare colour and surface characteristics among formulations. Throughout the manuscript, “nominal Ag loading” refers to the formulation level used during film preparation; “AgNP-equivalent concentration” refers only to the SPR-derived optical estimate in the release test; “total Ag” is used only for direct elemental determinations; and dissolved Ag was not measured in the present study. The nominal Ag mass per 4 cm^2^ film piece was calculated from the precursor stoichiometry and the 600 cm^2^ casting area. The Ag-containing precursor contained 1 mmol Ag in 100 mL, equivalent to 107.87 mg Ag. Because the precursor was added gravimetrically, an approximate density of 1.0 g mL^−1^ was used for this nominal calculation; thus, 8.44, 16.88, and 25.32 g of precursor corresponded to approximately 9.10, 18.21, and 27.31 mg Ag in the respective 600 cm^2^ films. A 4 cm^2^ piece represented 1/150 of the cast area, giving nominal Ag masses of approximately 0.061, 0.121, and 0.182 mg per piece for the 50, 100, and 150 mg L^−1^ formulations, respectively. These values are calculated nominal quantities and not direct measurements of total Ag in the dried films.

### 4.2. UV-Vis Release Test and Kinetic Modelling

A simplified release test was performed to estimate the plasmonically active AgNP-equivalent fraction released from the dried alginate film after rehydration. For each AgNP-containing formulation (50, 100, and 150 mg L^−1^), a 0.1 g piece of dried film was immersed in 50 mL of distilled water at room temperature. UV–Vis spectra of the aqueous phase were recorded after 5, 15, 25, 35, 45, 60, 80, 1380, 1440, 1560 and 1620 min, and absorbance was evaluated at the AgNP surface plasmon resonance band centred at approximately 416 nm. The term “AgNP-equivalent concentration” denotes an optical estimate derived from the SPR absorbance relative to freshly synthesised calibration standards. It describes the plasmonically active fraction and should not be interpreted as total silver concentration or silver speciation. The calibration curve was prepared from a dilution series of freshly synthesised AgNP gel obtained by xylose-mediated reduction of AgNO_3_ in the presence of alginate. The relationship between absorbance and AgNP-equivalent concentration was described by linear regression as A_416_ = 0.115621c + 0.001429. Release was expressed as a percentage of the formulation-specific theoretical maximum concentration; for the 100 mg L^−1^ formulation, this value was 7.114 ppm.

The release data were fitted using the Weibull equation F(t) = F_∞_[1 − exp(−(t/τ)^β^)], where F(t) is the released fraction at time t, F_∞_ is the asymptotic released fraction, τ is the time-scale parameter, and β is the shape parameter. The quality of fit was evaluated using R^2^ and RMSE. The Weibull model was selected a priori as a flexible empirical function for describing the common rapid-rise-to-plateau profile across the three formulations. It was used for descriptive comparison rather than as the outcome of a model-selection procedure, and the fitted parameters were not used to assign a unique physical release mechanism.

### 4.3. Five-Week-Old Arugula Plants in Peat Substrate

Square film pieces (4 cm^2^) were placed, one per chamber, in 54-cell trays filled with standard peat substrate (KLASMANN Substrate TS1, Hortico, Wrocław, Poland, pH = 6, fertilizer content: 1.0 kg m^−3^, packaging: 210 L, weight approximately 80 kg). The volume of a single cell was 0.076 L. Each occupied tray cell constituted one experimental unit. The experiment comprised 25 experimental units, with five biological replicates per treatment. The five treatments were randomly assigned to the occupied tray cells. No experimental blocking was applied. The greenhouse experiment was conducted once as a single experimental run. The nominal Ag mass associated with each 4 cm^2^ film piece was approximately 0.061 mg for the 50 mg L^−1^ formulation, 0.121 mg for the 100 mg L^−1^ formulation, and 0.182 mg for the 150 mg L^−1^ formulation, as calculated from the precursor stoichiometry and casting area. Each occupied tray cell constituted one experimental unit. The experiment comprised 25 units, with five biological replicates per treatment. Arugula seeds were sown (10 seeds per tray cell) and top-watered daily with tap water (10 mL per cell); no additional fertilizers were added. The experiment was conducted in a greenhouse at 20 °C, under a 12/12 h light/dark photoperiod, for 5 weeks.

Experimental treatments: C—no film added to the substrate; Alg—alginate film without AgNPs; 50 mg L^−1^ AgNPs, 100 mg L^−1^ AgNPs and 150 mg L^−1^ AgNPs—alginate films with nominal AgNP loadings of 50, 100, and 150 mg L^−1^, respectively.

### 4.4. Determination of Soil Microorganism Abundance

Five grams of substrate were collected from the root zone of each experimental cell. The material was then suspended in 45 mL of sterile distilled water and vigorously shaken for 2 h on a laboratory shaker (250 rpm). The abundance of bacteria and microscopic fungi was determined by surface-plating serial dilutions of the aqueous substrate suspension onto agar media using the modified Koch dilution method [46]. Bacterial and fungal populations were determined using 2.5% enriched agar or 6.5% Sabouraud medium (BioMaxima, Wrocław, Poland), respectively. After 72 h incubation at 30 °C, the well-developed colonies were assessed macroscopically and counted, and abundance was expressed as colony-forming units per gram of dry substrate (CFU g^−1^ DW).

### 4.5. Silver Content and Stress Biomarkers in Plant Tissues

Silver content was determined using a validated atomic absorption spectrometry method with an electrothermal atomic absorption spectrometer (ET-AAS) cuvette (AA240Z) from Varian, Mulgrave, Victoria, Australia)) in accordance with PN-EN 14084:2004 [47]. Wet digestion was performed by the microwave method under pressure (MarsXPres from CEM Corporation, Matthews, NC, USA)) with a mineralization time of 35 min, using nitric acid (Suprapur® grade, Merck KGaA, Darmstadt, Germany, catalog no. 1.00441) in the amount of 10 mL per 0.5 g of sample. The sealed mineralization containers were designed to achieve the appropriate pressure at which the liquid’s boiling point is above 200 °C. Silver content was determined using the standard addition method, which involves adding known, increasing amounts of the analyte to the sample and plotting a signal–concentration relationship to eliminate errors resulting from matrix effects. As part of the quality control of the method, Certified Reference Materials NCS ZC 73009 (China National Analysis Center for Iron and Steel, Beijing, China)) were tested.

The content of L-ascorbic acid in plant material was assessed by the iodometric method as described by Ikewuchi and Ikewuchi [48]. A plant sample (1.25 g) was homogenized with 10 mL of 1% oxalic acid. After 30 min, 5 mL of filtered extract containing 1 mL of 1% starch was titrated with a solution of iodine in potassium iodide. Ascorbic acid present in the solution reacted regularly with iodine. The iodine remaining after oxidation of ascorbic acid in the sample formed a blue complex with starch, indicating the completion of the titration. The ascorbic acid content was expressed as mg 100 g^−1^ FW. Glutathione content was assessed using the method of Guri [49] with modifications. One gram of fresh leaves was chopped and homogenized in an ice bath (4 °C) with 10.0 mL of 0.5 mM EDTA (Ethylenediaminetetraacetic acid) dissolved in 3% TCA (Trichloroacetic acid). The obtained extract was then centrifuged at 13,968× *g* for 10 min at 4 °C. Next, 2 mL of the supernatant was mixed with 5 mL of K-phosphate buffer (pH = 7.0, 0.1 M) to adjust the pH of the solution to approximately 7.0. Following this, 2 mL of the mixture was transferred to a second tube, to which an additional 1 mL of K-phosphate buffer was added. To this was added 0.1 mL of Ellman’s reagent, 5,5-dithiobis(2-nitrobenzoic acid). In parallel, blanks were also prepared for each analytical sample in a similar manner, but with 1.1 mL of potassium phosphate buffer (0.1 M) and without Ellman’s reagent. Reduced glutathione concentration was determined by measuring absorbance at 412 nm using a Helios Beta UV-VIS spectrophotometer (Waltham, MA, USA). The concentration was determined based on the Ellman reaction and a glutathione standard curve. Glutathione content was expressed as μg g^−1^ FW.

Phenolic compounds were determined based on the Folin–Ciocalteu method described by Djeridane et al. [50]. Plant material (2 g) was mixed with 10 mL of 80% methanol and then centrifuged (3492× *g* for 10 min at 18 °C). Plant extracts (0.1 mL) were mixed with 2 mL of 2% sodium carbonate. After 2 min, Folin–Ciocalteu reagent (0.1 mL) mixed with deionized water (1:1 *v*/*v*) was added to the tubes. The mixture was incubated at room temperature (20 °C) in the dark for 45 min. Absorbance was measured at 750 nm using a Helios Beta UV-VIS spectrophotometer (Waltham, MA, USA). Total phenolic content was expressed as mg gallic acid equivalents g^−1^ FW.

Antioxidant activity was assessed using the Molyneux method [51]. Plant material (2 g) was mixed with 10 mL of 80% methanol and then centrifuged (3492× *g* for 10 min at 18 °C). The reaction mixture consisted of 0.1 mL of supernatant and 4.9 mL of 80% methanol solution with a concentration of 0.1 mM DPPH. The control was a solution containing 0.1 mL of 80% methanol and 4.9 mL of DPPH radical. All samples were mixed and incubated for 15 min in the dark at room temperature. Then, the absorbance of all samples was measured at a wavelength of 517 nm using a Helios Beta UV-VIS spectrophotometer (Waltham, MA, USA). DPPH radical scavenging activity was determined using the formula DPPH [%] = [(A_0_−A_1_)/A_0_] × 100, where A_0_ was the absorbance of the control solution and A_1_ was the absorbance of the sample solution after incubation with DPPH. Antioxidant activity was expressed as %DPPH.

The assessment of peroxidase activity was based on the oxidation of p-phenylenediamine to phenazine by peroxidase using hydrogen peroxide. For analysis, 2 g of plant material was used, which was cold homogenized in a mortar with 10 mL of 0.05 M phosphate buffer, pH = 6.2, and then centrifuged (3492× *g* for 15 min at 4 °C). Samples were collected: 2 mL of supernatant, 2 mL of the same phosphate buffer, 0.2 mL of 1% p-phenylenediamine solution, and 0.2 mL of 0.1% hydrogen peroxide solution, which initiated the reaction. Absorbance was measured at 485 nm after 60 and 120 s of hydrogen peroxide addition using a Helios Beta UV-VIS spectrophotometer (Waltham, MA, USA). For each measurement sample, a blank sample was prepared, which contained 2 mL of supernatant, 2.2 mL of buffer and 0.2 mL of p-phenylenediamine solution. Peroxidase activity was expressed as the change in absorbance per minute per gram of fresh weight (U g^−1^ FW). One unit of enzyme activity (U) was defined as an increase in absorbance of 0.1 per minute.

### 4.6. Photosynthetic Pigments Content in Plant Tissues

The content of photosynthetic pigments chlorophyll *a*, chlorophyll *b*, and carotenoids was assessed with the method of Lichtenthaler and Wellburn [52]. Fresh leaves (0.1 g) were thoroughly mixed with 25 mL of 80% (*v*/*v*) acetone, with 3 mg of magnesium carbonate added as a stabilizer. The samples were then capped and left to incubate in the dark for 30 min. The resulting suspension was filtered and absorbance measurements were performed at wavelengths of 646, 663, and 470 nm using a Helios Beta UV-VIS spectrophotometer (Waltham, MA, USA) to determine the concentrations of chlorophyll *a*, chlorophyll *b* and total carotenoids, respectively.

### 4.7. Protein, Dietary Fiber and Sugar Content in Plant Tissues

Crude protein concentration was assessed using the Dumas method according to PN-EN ISO 16634-1:2008 [53] using a TruSpec N nitrogen analyzer (LECO Corporation, St. Joseph, MI, USA). The weighed sample (approximately 0.5 g) was placed in an aluminum capsule in an automatic feeder. The sample was then transferred to a reactor, where it was combusted in the presence of oxygen at 850 °C. The organic compounds were decomposed into carbon oxides, water, nitrogen oxides, and molecular nitrogen. The gases passed through a copper reduction column, where they were reduced to molecular nitrogen at 950 °C. Water and carbon dioxide were removed. The remaining nitrogen was quantified using a thermal conductivity detector TruSpec N (LECO Corporation, St. Joseph, MI, USA). The amount of nitrogen was then recalculated into protein content using a conversion factor of 6.25. The calibration standard was ethylenediaminetetraacetic acid (Fluka, Solstice Advanced Materials, NJ, USA). As a certified reference material, NCS ZC73013 Spinach (China National Analysis Center for Iron and Steel, Beijing, China) was used. Crude protein content was expressed as g 100 g^−1^ DW. Dietary fiber content was measured using an enzymatic-gravimetric method combining the principles of AOAC methods: 2002.02, 985.29, 991.43, 2001.03, and 2009.01 [54] with a commercially available assay kit (Megazyme, Lansing, MI, USA). Before analysis, the plant material was dried, ground, and thoroughly homogenized. Duplicate aliquots of approximately 1.0 g were analyzed. The samples were incubated with pancreatic α-amylase and amyloglucosidase for 4 h at 37 °C in tightly sealed 250 mL bottles with continuous stirring or shaking. During this step, non-resistant starch was solubilized and hydrolyzed to D-glucose and trace amounts of maltose. The reaction was terminated by adjusting the pH to 8.2, followed by heating to approximately 95 °C. Four volumes of 95% ethanol were subsequently added to the incubation mixture to precipitate the high-molecular-weight soluble dietary fiber fraction. The samples were then filtered under vacuum through pre-weighed crucibles containing Celite. The residues were washed successively with 78% ethanol, 95% ethanol, and acetone, dried at 105 °C to constant weight, cooled in a desiccator, and weighed. Dietary fiber content was calculated according to the manufacturer’s instructions and expressed as g 100 g^−1^ DW.

Simple sugars were determined using the anthrone colorimetric method [55]. Analysis was performed by extracting 1 g of fresh plant tissue in 50 mL of 80% ethanol. The mixture was heated to 100 °C to extract soluble sugars. A 4 mL amount of anthrone reagent (2 g of anthrone dissolved in 100 mL of concentrated H_2_SO_4_) was then added to the prepared extract. The samples were incubated for 10 min at 100 °C and then cooled to room temperature. Absorbance was measured at 625 nm using a Helios Beta UV-VIS spectrophotometer (Waltham, MA, USA). Sugar content was estimated using a standard curve. The sugar content is expressed in %.

### 4.8. Statistical Analysis

The statistical analysis was carried out by performing a one-way analysis of variance (ANOVA), with treatment as the fixed factor (C, Alg, 50 mg L^−1^ AgNPs, 100 mg L^−1^ AgNPs, and 150 mg L^−1^ AgNPs). Each treatment consisted of five biological replicates (*n* = 5). Mean values were compared using Fisher’s least significant difference (LSD) test at *p* ≤ 0.05 using the Statistica 13.3 software (TIBCO Software Inc., Palo Alto, CA, USA). This procedure was selected because the number of treatment levels was limited and the comparisons were based on predefined, biologically relevant treatment effects. Under these conditions, Fisher’s protected LSD provides sufficient sensitivity while the preceding omnibus ANOVA reduces the risk of identifying spurious differences. Results are presented as means ± standard error (SE). In the figures, different letters indicate statistically significant differences among treatments, whereas the absence of letters indicates no statistically significant differences at *p* ≤ 0.05.

## 5. Conclusions

Using sodium alginate as a biodegradable carrier and xylose as a reducing agent enabled the preparation of AgNP-containing films. Under the tested nominal loadings, the films did not significantly affect the abundance of culturable rhizosphere bacteria or fungi. In five-week-old arugula plants, the responses were selective across the measured endpoints rather than uniformly adverse: silver accumulated in the tissues, phenolic content decreased and antioxidant activity varied with treatment, whereas peroxidase activity, ascorbic acid, photosynthetic pigments, glutathione and total sugars remained largely unchanged. The aqueous release experiments performed for all three AgNP-containing formulations showed closely comparable profiles, with a rapid initial increase in the plasmonically active AgNP-equivalent signal followed by a slower approach to a quasi-plateau. These UV–Vis-derived values describe an optically detectable AgNP-equivalent fraction and should not be interpreted as total silver release, silver speciation or proof of unchanged nanoparticle stability. Overall, the results provide an initial assessment of the response of non-target plant and culturable rhizosphere microorganisms to a biodegradable AgNP carrier system. Further work should include ionic-silver controls, silver speciation, release measurements in peat, broader microbial-community analysis and more representative microscopic sampling before the environmental safety or application potential of the material can be established. The findings do not support the use of the tested films as plant-growth-promoting treatments or permit the derivation of a field application rate. Their practical significance lies instead in demonstrating that a biodegradable material with potential antimicrobial functionality can release biologically available silver under plant–substrate conditions without causing broad adverse changes across the measured short-term endpoints, while still producing silver accumulation and selective biochemical responses in an edible plant.

## Figures and Tables

**Table 1:** Descriptive SEM image-analysis summary of particle-like objects observed in selected micrographs of AgNP-containing alginate films.

Film Formulation	*n* *	Mean Equivalent Diameter ± SD (nm)	Image-Based Dispersion Index, (SD/mean)^2^	Mean Circularity	Mean Aspect Ratio	Maximum Feret Diameter (nm)
50 mg L^−1^ AgNPs	36	7.42 ± 1.40	0.036	0.819	2.06	23.80
100 mg L^−1^ AgNPs	60	7.93 ± 1.59	0.040	0.854	1.94	28.18
150 mg L^−1^ AgNPs	108	8.57 ± 1.79	0.044	0.843	1.96	33.17

* *n* denotes the number of clearly distinguishable particle-like objects included in the analysis of one selected SEM micrograph for each formulation; it does not represent nanoparticle concentration in the film. The image-based dispersion index was calculated as (SD/mean)^2^ and is a descriptive proxy; it should not be interpreted as a polydispersity index determined by dynamic light scattering. Circularity ranges from 0 to 1, where 1 represents a perfect circle. The reported values should be interpreted as field-specific descriptive estimates.

**Table 2:** Comparative kinetic parameters and release metrics for the plasmonically active AgNP-equivalent fraction released from AgNP-containing alginate films.

Formulation	Final AgNP-Equivalent Concentration at 1620 min (ppm)	Experimental Release at 1620 min (%)	*F_∞_* (%)	*τ* (min)	*β*	R^2^	RMSE (%)
50 mg L^−1^	4.36	69.65	68.27	9.11	1.385	0.9908	1.23
100 mg L^−1^	5.01	70.46	67.46	8.92	1.30	0.9865	1.42
150 mg L^−1^	5.46	70.57	69.41	8.85	1.400	0.9975	0.63

Calibration equation: A416 = 0.115621c + 0.001429 (R^2^ = 0.99993). Release values are expressed as AgNP-equivalent concentrations of the plasmonically active fraction and should not be interpreted as total silver release or silver speciation. The Weibull model was used as an empirical descriptor for comparative purposes.

**Table 3:** Mass distribution of chemical reagents used during the synthesis of silver nanoparticles in alginate films. Individual alginate films: Alg—alginate film without silver nanoparticles; 50 mg L^−1^ AgNPs, 100 mg L^−1^ AgNPs and 150 mg L^−1^ AgNPs—alginate films with nominal loadings of AgNPs at 50, 100 and 150 mg L^−1^.

Samples	Water [g]	Sodium Alginate [g]	Solution with Ag^+^ [g]	Glycerol [g]	Xylose Solution (4%) [g]	Total Mass [g]	Nominal AgNP Loading [mg L^−1^]
Alg	195.50	3.00	0.00	1.50	0.00	200.00	0.00
50 mg L^−1^ AgNPs	173.39	3.00	8.44	1.50	13.67	200.00	50.00
100 mg L^−1^ AgNPs	151.27	3.00	16.88	1.50	27.35	200.00	100.00
150 mg L^−1^ AgNPs	129.16	3.00	25.32	1.50	41.02	200.00	150.00

## Data Availability

The raw data supporting the conclusions of this article will be made available by the authors on request.
